# The *grooveless* gene encodes a single von Willebrand factor type C domain protein necessary for proper formation of the scutoscutellar sulcus in *Drosophila melanogaster*

**DOI:** 10.17912/micropub.biology.002111

**Published:** 2026-03-20

**Authors:** Kevin R. Cook, Stephanie E. Mauthner

**Affiliations:** 1 Bloomington Drosophila Stock Center, Department of Biology, Indiana University Bloomington, IN, USA

## Abstract

We show that disruption of the *CG31997* gene on the fourth chromosome of *Drosophila melanogaster* by *grooveless* mutations results in loss of the scutoscutellar sulcus, the hinge-like region between the two major divisions of the fly thorax necessary for normal wing movement in flight. This gene belongs to a family of arthropod secreted proteins with a single von Willebrand factor type C domain, and our study expands the biological roles of these enigmatic proteins to include morphogenesis.

**Figure 1. The phenotypic and molecular characterization of the grooveless gene:**
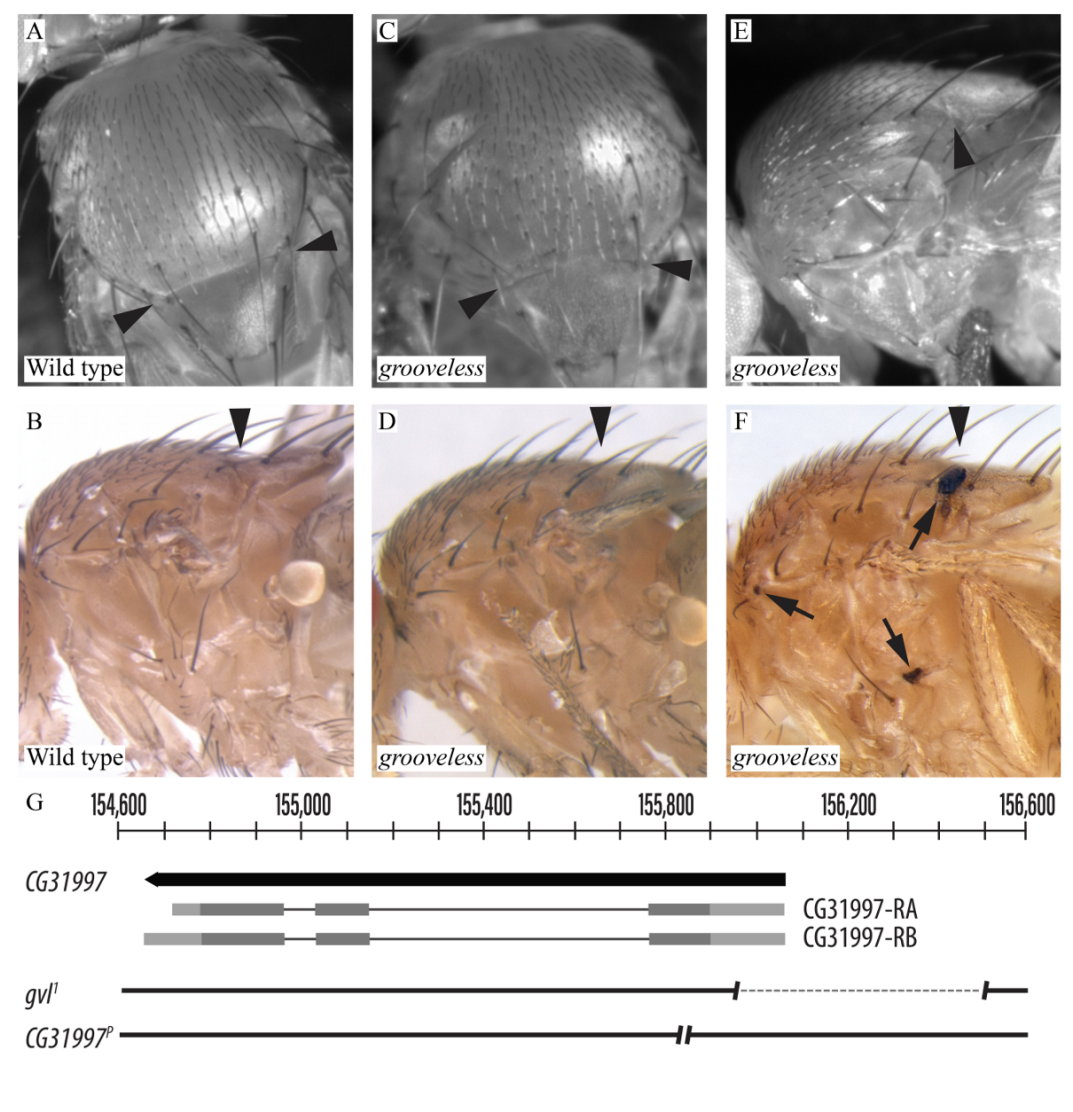
(A) Dorsal view of a wild type fly showing the marked indentation of the dorsal thorax between the scutum and scutellum forming the scutoscutellar sulcus (marked by arrowheads in all panels). (B) Lateral view of a wild type fly with its left wing removed to demonstrate the typical depth of a sulcus. (The fly in panel A came from stock 6599; the fly in panel B from stock 64349.) (C) Dorsal view of a *grooveless* ( *gvl* ) mutant (a *CG31997 ^P^* homozygote) showing a nearly complete absence of the sulcus. (D and E) Lateral views of *grooveless* mutants (a *gvl ^1^* homozygote in D and a *CG31997 ^P^* homozygote in E) with very shallow sulci. (Panels C and E show the same fly.) (F) Lateral view of another *CG31997 ^P^* homozygote with clotted, melanized hemolymph (arrows) at the lateral end of an abnormally shallow sulcus, below the humeral bristles and above and posterior to the sternopleural bristles. (G) Whole-genome sequencing of *gvl ^1^* homozygotes revealed a 545 bp deletion removing 5’ UTR and adjacent intergenic sequences. Previous work by the Fourth Chromosome Resources Project demonstrated that *CG31997 ^P^* is a five bp deletion likely causing frameshift truncation of the *CG31997* protein. &nbsp; &nbsp;

## Description

In 1933, Calvin Bridges discovered a recessive mutation on the *Drosophila melanogaster* fourth chromosome that reduces or eliminates the scutoscutellar sulcus—the transverse groove separating the larger subdivision of the notum, the scutum, from the smaller subdivision, the scutellum. He named the mutated gene *grooveless* ( *gvl* ) for this phenotype (Bridges 1935). Figures 1A and B show wild type thoraces while Figures 1C–E show the *grooveless* phenotype. Bridges noted that clotted hemolymph is sometimes seen in mutants at the lateral ends of sulci and near the sternopleural and humeral bristles ( Figure 1F ). The original mutation, *gvl ^1^* , has been used as a recessive visible marker for tracking the fourth chromosome and mapping mutations, but has otherwise received little attention—even though flexion between the scutum and scutellum at the scutoscutellar sulcus is a part of normal flight (Boettinger and Furshpan 1952; Miyan and Ewing 1985).

&nbsp;

Sturtevant (Sturtevant 1951) and Hochman (Hochman et al. 1964) localized *gvl* to the proximal part of the fourth chromosome. Unpublished work by the DrosDel consortium (Ryder et al. 2007) documented in FlyBase (Öztürk-Çolak et al. 2024) further localized *gvl* to a genomic interval encompassed by *Df(4)ED6366* containing eight protein-coding genes and three noncoding RNA genes *.* Hochman and colleagues showed that *gvl ^1^* complemented all mutations in loci necessary for viability identified in their extensive characterization of the fourth chromosome (Hochman 1976). Because they isolated mutations in nearly every fourth chromosome gene that could be mutated to lethality (Weasner et al. 2025), the null phenotype of *gvl* is unlikely to be lethality.

*&nbsp;*

We verified the DrosDel mapping of *gvl ^1^* to *Df(4)ED6366* and complementation tested *gvl ^1^* against loss-of-function mutations in the eight protein-coding genes within the region of *Df(4)ED6366* : *Ank ^A^ , Ank ^J^ , anne ^D^ , CG32006 ^CR70476-kG4^ , CG31997 ^CR70475-TG4.1^ , CG33978 ^B^ , Arl4 ^D^ , Abcd1 ^A^ , Abcd1 ^K^* and *pan ^ciD^* (though Hochman (1976) demonstrated that *gvl ^1^* was not an allele of *pan* ). We found that *gvl ^1^* and *Df(4)ED6366* failed to complement *CG31997 ^CR70475-TG4.1^* and that flies homozygous for this allele show shallow scutoscutellar sulci. Subsequently, another *CG31997* allele ( *CG31997 ^P^* ) became available from the Fourth Chromosome Resource Project (Weasner et al. 2025). It is a frameshift mutation (deletion of bases 4:155817 through 155821) predicted to add an arginine after residue 28 of the 147-amino acid *CG31997* protein before truncating the protein; consequently, it is likely a null allele. It failed to complement *gvl ^1^* , *CG31997 ^CR70475-TG4.1^* and *Df(4)ED6366* as well as a derivative of *CG31997 ^CR70475-TG4.1^* called *CG31997 ^CR70475-DH.PT-GFSTF.1^* , and it showed the abnormal sulcus phenotype when homozygous. Interestingly, many flies from noncomplementing crosses involving *CG31997 ^P^* and many flies homozygous for *CG31997 ^P^* showed scutellar, humeral and/or sternopleural clots—phenotypes we did not see as frequently in stocks with other alleles or in crosses involving alleles other than *CG31997 ^P^* . Since hemolymph leakage seems particularly deleterious, we suspect suppressors of this phenotype accumulate in *gvl* stocks. Heteroallelic flies with *gvl ^1^* , *CG31997 ^CR70475-TG4.1^* or *CG31997 ^CR70475-DH.PT-GFSTF.1^* combined with *CG31997 ^P^* and flies with *CG31997 ^P^* combined with *Df(4)ED6366* were capable of flight, but we did not assess flight duration or efficiency, nor the details of thoracic movement.

&nbsp;

Whole-genome sequencing of Bloomington Drosophila Stock Center stocks 640 and 650 showed that *gvl ^1^* is a 545 bp deficiency (bases 4:155948 through 4:156492 deleted) removing 72% of the *CG31997* 5’ UTR and 29% of the adjacent *CG31997–CG33978* intergenic region ( Figure 1G ). Thirty-three bp of unknown origin (AAAAAAAAATAAAATTAAATACAAATTTAATTG) are inserted at the breakpoint junction.

&nbsp;

*CG31997* , which we now call *grooveless* , encodes a protein with a single von Willibrand factor type C (SVWC) domain and a cell export signal, but no other known protein motifs (Sheldon et al. 2007). Proteins with a SVWC domain appear to be restricted to arthropods, but the biological functions of very few of these proteins are known (Labropoulou et al. 2024). Of the fourteen *D. melanogaster* genes annotated as SVWC genes in FlyBase (Öztürk-Çolak et al. 2024), only *Vago* has been characterized in depth. It encodes a protein with interferon-like activities in immunity (Xia et al. 2025). Our molecular identification of *gvl* represents the first example of a SVWC gene involved in morphogenesis and offers new opportunities to study thoracic development and the role of the hinge-like movement of the scutum and scutellum in the mechanics of flight.

&nbsp;

&nbsp;

&nbsp;

## Methods

The stocks used in complementation tests are listed in the Reagents Table. More information about these stocks may be obtained via the website for the Bloomington Drosophila Stock Center (https://bdsc.indiana.edu/). Genetic nomenclature and gene models reflect FlyBase release 2025_5 (Öztürk-Çolak et al. 2024). DNA was extracted from frozen samples of 15 adult flies using standard protocols and sequenced as single-end reads on the Ultima Genomics platform by the University of Minnesota Model Organism Sequencing Service (https://genomics.umn.edu/service/model-organism-sequencing). DNA yields averaged 1,082 ng per sample with mean NanoDrop A260/280 and A260/230 ratios of 1.83 and 0.79. For stocks 640 and 650, mean genome coverages were 57x and 77x and alignment rates to the *D. melanogaster* Release 6 reference genome assembly were 94.17% and 87.48%, respectively.

## Reagents

**Table:** 

Stock number	Genotype	RRID	Reference
640	* ci ^1^ gvl ^1^ bt ^1^*	RRID:BDSC_640	Bridges 1935
641	* ci ^1^ gvl ^1^ ey ^R^ sv ^n^*	RRID:BDSC_641	Bridges 1935
650	* gvl ^1^*	RRID:BDSC_650	Bridges 1935
1634	Dp(1;Y)y ^+^ /y ^1^ ; cn ^1^ l(2)* ^*^ /SM1; e ^1^ ; gvl ^1^	RRID:BDSC_1634	Bridges 1935
6599	* y ^1^ w ^67c23^*	RRID:BDSC_6599	&nbsp;
8067	* w ^1118^ ; Df(4)ED6366, P{w ^+mW.Scer\FRT.hs3^ =3'.RS5+3.3'}Abcd1 ^ED6366^ pan ^ED6366^ /Dp(2;4)ey ^D^ , Ablp ^eyD^ : ey ^D^*	RRID:BDSC_8067	Ryder et al. 2007
9422	* w ^1118;^ Df(4)ED6369, P{w ^+mW.Scer\FRT.hs3^ =3'.RS5+3.3'}ED6369/l(4)102EFf ^1^*	RRID:BDSC_9422	Ryder et al. 2007
64349	*Canton-S*	RRID:BDSC_64349	&nbsp;
97181	y ^1^ w ^*^ ; TI{GFP ^3xP3.cLa^ =CRIMIC.TG4.1}CG31997 ^CR70475-TG4.1^	RRID:BDSC_97181	Lee et al. 2018
97333	y ^1^ w ^*^ ; TI{GFP ^3xP3.cLa^ =SA-KozakGAL4}CG32006 ^CR70476-kG4^ /In(4)ci ^D^ , ci ^D^ pan ^ciD^	RRID:BDSC_97333	Kanca et al. 2022
97745	* y ^1^ w ^1118^ ; TI{DH.1}CG31997 ^CR70475-DH.PT-GFSTF.1^*	RRID:BDSC_97745	Stinchfield et al. 2024
602194	* y ^1^ w ^1118^ ; TI{TI}FRT101F Ank ^A^*	RRID:BDSC_602194	Weasner et al. 2025
602199	* y ^1^ w ^1118^ ; TI{TI}FRT101F Abcd1 ^A^*	RRID:BDSC_602199	Weasner et al. 2025
605312	* y ^1^ w ^1118^ ; TI{TI}FRT101F anne ^D^ / In(4)ci ^D^ , ci ^D^ pan ^ciD^*	RRID:BDSC_605312	Weasner et al. 2025
605963	* y ^1^ w ^1118^ ; TI{TI}FRT101F CG33978 ^B^ / In(4)ci ^D^ , ci ^D^ pan ^ciD^*	RRID:BDSC_605963	Weasner et al. 2025
605964	* y ^1^ w ^1118^ ; TI{TI}FRT101F Abcd1 ^K^ / In(4)ci ^D^ , ci ^D^ pan ^ciD^*	RRID:BDSC_605964	Weasner et al. 2025
605976	y ^1^ w ^*^ ; TI{TI}FRT101F Ank ^J^	RRID:BDSC_605976	Weasner et al. 2025
606073	* y ^1^ w ^1118^ ; TI{TI}FRT101F Arl4 ^D^*	RRID:BDSC_606073	Weasner et al. 2025
606864	y ^1^ w ^*^ ; TI{TI}FRT101F CG31997 ^P^	RRID:BDSC_606864	Weasner et al. 2025
